# Interferon‐Mediated Regulation of Memory B Cell Identity and Function

**DOI:** 10.1111/imr.70161

**Published:** 2026-08-22

**Authors:** Alana Kirn, Kim L. Good‐Jacobson

**Affiliations:** ^1^ Department of Biochemistry and Molecular Biology, Immunity Program, Biomedicine Discovery Institute Monash University Clayton Australia

## Abstract

Memory B cells are essential for long‐lasting immunity induced by both encountered pathogens and vaccines. Functional diversity within the memory B cell pool is established through interactions between memory B cells and the environment in which they develop and reside. This review will discuss novel insights into the role of cytokines encountered in the microenvironment in modulating memory B cell transcriptomic, epigenetic, and phenotypic identity. Specifically, it will focus on how interferons can prime unique memory B cell subpopulations upregulated in chronic viral infection, autoimmunity, and cancer. Understanding how memory B cell identity is established and maintained in interferon‐primed memory B cell subsets, and how memory B cell phenotype relates to function, may help advise novel vaccine design and diagnostic procedures for a wide range of immunopathologies.

## 1 Introduction

A fundamental strength of the humoral immune system is its ability to mount robust secondary immune responses upon re‐exposure to a previously encountered pathogen. This immune memory is governed by long‐lived plasma cells and memory B cells. Long‐lived plasma cells are a terminally differentiated B cell subset that localize to the bone marrow and constitutively secrete antibodies in the absence of an ongoing antigen burden [1]. Memory B cells are a quiescent population of antigen‐experienced B cells that can rapidly reactivate upon antigen exposure to drive rapid pathogen clearance [2]. The generation and persistence of memory B cells following immunization is a critical factor that underlies the success of vaccines and the prevention of serious disease in vaccinated people upon infection [3].

Examining the memory B cell pool more closely unveils the complexity of this population. Memory B cells are highly heterogeneous in origin [4], phenotype [5, 6, 7] and epigenomic identity [8, 9, 10]. The developmental pathways and functional capabilities of some memory B cell subsets are well‐characterized, particularly those emerging from canonical humoral immune responses to vaccination and acute viral infection. The distinction between germinal center‐derived and extrafollicular memory B cells, the role of isotype switching in generating memory B diversity and the correlation between surface marker phenotype and memory B cell fate in secondary immune responses are all recognized in the literature (reviewed elsewhere [11, 12, 13]). However, memory B cell populations associated with dysregulated immune contexts, including chronic viral infection [14, 15], cancer [16, 17] and autoimmunity [18, 19] are not as well understood. The phenotype of responding memory B cells and their connection to “canonical” subsets, their functional capabilities and the molecular drivers underlying their emergence are all areas of ongoing research.

Recent work into characterizing B cells in dysregulated immune responses has demonstrated that the memory B cell pool is diversified through interactions between the memory B cells and the wider immune landscape in which they develop and reside [20, 21]. In this review, we will discuss new insights into how memory B cell identity is established and maintained, with a focus on the role of interferon in driving the emergence of novel memory B cell subsets. Altered interferon signaling, and the subsequent emergence of altered memory B cell populations, is a shared hallmark of a range of diverse pathologies with limited clinical preventative strategies or treatment options [22, 23, 24, 25, 26, 27]. Understanding the molecular mechanisms that drive the emergence of interferon‐primed memory B cell populations, their functional capabilities, and how refractive they may be to remodeling by therapeutic intervention may provide the key to designing novel vaccines, treatments, and diagnostic procedures for patients.

## 2 Memory B Cell Formation

### 2.1 T Cell‐Dependent Memory B Cell Development

Memory B cells are generated from naïve B cells during primary antigen encounter [28]. In primary responses, naïve B cells are activated upon binding of their BCR to its cognate antigen [28]. Activated B cells subsequently migrate to the T‐B cell zone border and present antigen via MHC‐II to CD4^+^ T cells [28]. This interaction, in turn, promotes upregulation of CD40L and cytokine production by T cells, driving B cell proliferation and differentiation [4, 28]. Post‐activation, memory B cell differentiation can occur through two distinct pathways.

The majority of memory B cells in the human memory B cell compartment are derived from germinal centers: transient structures that form in the follicle and serve to enhance the affinity and efficacy of B cell‐mediated immune responses through affinity maturation [4]. In activated B cells, the germinal center reactions are initiated upon upregulation of the transcription factor Bcl6 downstream of BCR signaling [29, 30, 31]. Germinal center B cells cycle between two spatially distinct zones: the light zone and the dark zone [4]. In the germinal center dark zone, the enzyme activation‐induced deaminase (AID) deaminates cytidine residues in the VDJ regions of the Ig gene, introducing point mutations into the Ig variable genes to drive somatic hypermutation of rapidly proliferating B cells [32, 33, 34]. This generates a diverse BCR repertoire within the B cell pool [32, 33]. Germinal center B cells proceed to enter the light zone, in which high‐affinity B cells are subsequently selected to survive via positive selection, while low‐affinity clones die by apoptosis [35]. This selection process is facilitated by B cell interactions with follicular dendritic cells that maintain antigen reservoirs in the germinal center [35, 36, 37, 38], and a population of CD4^+^ follicular T helper cells [39, 40]. Throughout these reactions, high‐affinity germinal center B cells upregulate Blimp‐1 and the plasma cell program, driving plasma cell differentiation [41, 42]. Likewise, upregulation of the memory B cell program in germinal center B cells drives the formation of long‐lived, high affinity memory B cells [43]. The precise molecular determinants underlying this fate decision are incompletely understood, and a single transcriptional regulator of memory B cell differentiation has not yet been defined. However, several transcriptional changes have been associated with pre‐memory formation in the germinal center and suppression of terminal plasma cell differentiation, including Bcl6 downregulation and suppression of mTORC1 and c‐Myc activity by Bach2 [44, 45].

A population of memory B cells can also form independent of the germinal center. These memory B cells first develop within 3 days post‐antigen exposure and continue to differentiate over the course of the immune response [4, 46]. Extrafollicular memory B cells are primarily IgM^+^, with only 1%–3% undergoing class switch recombination, and as expected, harbor fewer somatic hypermutations than their germinal center‐derived counterparts [4]. Several environmental cues have been implicated in preferentially driving extrafollicular plasmablast differentiation over germinal center entry, including high B cell receptor signaling strength [47] and transcriptional regulation of IRF4 expression [48]. Comparatively, the factors that promote germinal center independent memory B cell formation over plasmablast differentiation are poorly defined. Recently, Xu et al. have reported a role for Notch2 in pre‐germinal center memory B cell formation, with selective deletion of Notch2 in mature B cells reducing germinal center‐independent memory B cell differentiation post‐immunization [49]. Mechanistically, Notch2 signaling enhances BCR signaling and CD21 expression, and conversely, BCR antagonism promotes Notch2 expression [49]. This implicates both Notch2 and BCR signaling in germinal center‐independent memory B cell formation; however, the cellular and microenvironmental determinants of this fate decision remain under investigation. In immunization, low antigen availability can also preferentially drive memory B cell formation over plasmablast differentiation [46]. This suggests that there is a role for antigen load in extrafollicular memory B cell differentiation [46].

### 2.2 Memory B Cell Development in the Absence of T Cell Help

B cell activation can occur in the absence of CD4^+^ T cell interaction, driven by BCR crosslinking, co‐receipt of BCR‐mediated signals and toll‐like receptor (TLR) stimulation, and cytokine signaling through IL1R or IL18R [50]. These activated B cells have been shown to have the capacity to form memory. Memory B cells generated independent of T cell help are quiescent and persist for at least 120 days post‐infection [51]. Characterization of these cells has been largely limited to immunodeficient mouse models lacking T cells, in which they can be reactivated to produce plasmablast and antibody responses in secondary infection [50]. Limited studies have been performed to dissect the role that T‐independent memory B cells may play in immunocompetent hosts; however, there is evidence that their responsiveness to antigen stimulation may be inhibited by antibody feedback from antibodies produced in T‐dependent responses [51, 52]. Therefore, their overall role in the immune system is not well characterized.

### 2.3 Memory B Cells Can Be Divided Into Subpopulations

Beyond their diverse origins and developmental pathways, memory B cells are highly heterogeneous in phenotype and function. Each of these characteristics can be used to divide memory B cells into distinct subpopulations. However, the classification of memory B cells into these subpopulations is context‐dependent and not consistent between humans and mouse models [53]. Common classification schemes used in the literature are outlined in Table 1.

**TABLE 1:** Memory B cell classification schemes in humans and mice.

Memory B cell subset	Model	Characteristic features
“Canonical” memory B cells	Human	Express CD27 (human) and CD21 (human and mice) [54, 55, 56] Poised to differentiate into plasmablasts in secondary antigen encounter [57, 58]
	Mouse	
Switched and unswitched memory B cells	Human	Characterized according to surface immunoglobulin isotype [59, 60, 61, 62, 63] Switched memory B cells preferentially differentiate into antibody‐secreting cells in secondary infection [59, 60, 61] Unswitched memory B cells may reseed germinal centers [59, 61] or differentiate into antibody‐secreting cells [59, 61, 62, 63] The relative frequencies of switched and unswitched memory B cells can modulate cytokine production in SARS‐CoV2 infection [64]
	Mouse	
Double negative (DN) memory B cells	Human	Downregulate CD27 and IgD [65] Memory B cells are classified as DN1, DN2 and DN3 [65] DN1 memory B cells are CD21^+^CXCR5^+^CD11c^−^, and may be a IgD^−^CD27^+^ memory B cell precursor [24, 65, 66] DN2 memory B cells are CD21^−^CXCR5^−^CD11c^+^ and are poised for plasmablast differentiation with TLR7 signaling [24] DN3 memory B cells are CXCR5^−^CD21^−^CD11c^−^ and may be a DN2 precursor [67]
Double positive/negative memory B cells	Mouse	Memory B cells are classified by their surface expression of CD80, PD‐L2 and CD73 [5, 68, 69] Double positive (CD80^+^PD‐L2^+^) memory B cells are associated with antibody‐secreting cell formation in secondary infection [5, 69] Double negative (CD80^−^PD‐L2^−^) memory B cells are associated with secondary germinal center formation [5, 69] Increased CD73 expression in memory B cells is indicative of higher mutation rates [68]
“Atypical” B cells (also referred to as age‐associated or tissue‐like)	Human	Expression of CD11c and transcription factor T‐bet, and downregulation of CD21 [22, 25, 26, 70, 71] Expanded in dysregulated immune environments [22, 25, 26, 70, 71]
	Mouse	

To move toward a universal classification scheme for memory B cells, it is important to identify analogous memory B cell populations in different immune contexts that have similar functions. By considering the microenvironmental and molecular drivers of different memory B cell subsets, shared populations may be more clearly and definitively recognized.

## 3 Cytokines in the Microenvironment Can Modulate Memory B Cell Function

Memory B cells and their precursors communicate extensively with a complex network of immune and non‐immune populations in the microenvironment [72]. A key aspect of this communication is the synthesis, secretion, and receipt of cytokines [73, 74, 75, 76]. The rapid induction and tight regulation of cytokine responses are important factors underlying the success of pathogen clearance, with dysregulated cytokine production associated with increased pathogen burden and poor immune responsiveness [77, 78, 79, 80]. The specific cytokines produced, their concentrations, and the kinetics of their production are highly variable. Individual genetics, environmental factors such as seasonality and the microbiome, and, where applicable, pathogen‐specific factors such as pattern associated molecular patterns and pathogen load all influence the induced cytokine response [81, 82]. Cytokines modulate immune activity in a broad range of target populations in cytokine‐ and cell‐specific manners [83]. Accordingly, differences in cytokine signaling in the microenvironment can promote divergent immune responses in different immune contexts.

### 3.1 Cytokines Modulate B Cell Fate and Memory B Cell Function

Several cytokines are implicated in B cell differentiation, and in the modulation of B cell function (summarized in Table 2). Notably, cytokines can act on memory B cells and their precursors to regulate (1) memory B cell development within and departure from the germinal center [84, 85, 86, 87], (2) class switch recombination and affinity maturation [88, 89, 90, 91], and (3) the phenotype and function of memory B cell populations [8, 9, 18, 92, 93, 94, 95]. Correspondingly, receipt of cytokines is both required for memory B cell formation and underlies the emergence of distinct memory B cell subsets.

**TABLE 2:** The role of cytokines in memory B cell formation and identity.

Cytokine	Role in memory B cell formation and identity
IL‐2	Promotes an immunoregulatory phenotype in memory B cells [92, 93]
IL‐4	Promotes B cell survival and class‐switch recombination [88]
	Acts antagonistically to IL‐21 in the germinal center to downregulate Bcl6 and favor memory B cell differentiation over the plasma cell fate [84]
	Implicated in affinity‐based memory B cell selection [84]
IL‐6	IL‐6 blockade reduces B cell receptor mutational frequency [90, 91]
	Dampens activation in memory B cells derived from rheumatoid arthritis patients [91, 96]
IL‐9	Induces ZBTB18 in memory B cell precursors within the germinal center, to promote survival, cell‐cycle exit and germinal center egress [87]
	IL‐9R‐deficient mice fail to mount strong antibody‐mediated recall responses upon secondary infection [97]
	Treatment with recombinant IL‐9 in vitro restores class switch memory B cell formation in combined variable immunodeficiency patients [89]
IL‐10	Produced by regulatory B cells, to restrain immune activation and restore homeostasis [92, 93, 98]
	Enhances human B cell survival, proliferation, differentiation and isotype switching to IgG4 [99]
IL‐21	Promotes proliferation in activated B cells by accelerating cell cycle progression and fueling cyclic re‐entry of B cells [85, 86]
	Required for Bcl6 upregulation and the establishment of germinal center B cells [100]
	Induces antibody‐secreting cell production in human IgM‐ and isotype‐switched memory B cells [101]
	In the absence of IL‐21, memory B cell populations form in primary infection, but fail to respond robustly to secondary antigen encounter [102]
	Necessary for Type 2 (IgG1^+^CD23^+^IL‐4Rα^+^CD38^+^) memory B cell formation in mice: the precursor population for IgE plasma cells [103]
IL‐24	Modulates transcription factors involved in plasma cell differentiation to suppress the plasma cell fate [104]
TNFα	Dampens activation in memory B cells derived from rheumatoid arthritis patients [91]
Type I interferon	Synergizes with TLR stimulation to enhance T cell‐independent antibody responses [105]
	Drives the emergence of a distinct memory B cell subpopulation with unknown functional capabilities [8]
Type II interferon	Promotes class switching to IgG2 in humans and IgG2c in mice [106, 107, 108]
	Promotes T‐bet^+^ memory B cell formation [94, 95]

While memory B cell differentiation is not dependent on germinal center formation, limiting the B cell pool to those of extrafollicular origin can result in a functional deficit in recall responses [102]. Accordingly, memory B cell formation is influenced by cytokines required for germinal center establishment. In activated B cells, interleukin (IL)‐21 promotes proliferation by accelerating cell cycle progression and fueling cyclic re‐entry [85, 86], and BCL6 upregulation [100], to drive germinal center formation. Memory B cells can form in the absence of IL‐21 in primary infection, but these cells fail to respond robustly to secondary antigen encounter [102]. The process of affinity maturation and the subsequent generation of efficacious, high‐affinity memory B cells in the germinal center is also dependent on IL‐6, with IL‐6 blockade reducing B cell receptor mutational frequency of memory B cells [90, 91].

Exit from the germinal center as a plasma cell or memory B cell is dependent on the upregulation of the associated molecular program [44, 109]. Therefore, memory B cell differentiation is dependent on the receipt of cytokines to both downregulate the germinal center and plasma cell programs, and upregulate the memory B cell program [44, 109]. In the germinal center, IL‐4 and IL‐9 can both drive memory B cell formation [84, 87]. IL‐4 downregulates Bcl6, the master transcription factor of the germinal center, to favor memory B cell differentiation over germinal center maintenance and the plasma cell fate [84]. Similarly, IL‐9 promotes B cell survival, cell‐cycle exit and germinal center egress in germinal center memory B cell precursors, through the induction of ZBTB18 [87]. Furthermore, IL‐24 can directly modulate transcription factors involved in plasma cell differentiation, to suppress the plasma cell fate [104]. Beyond the requirement for cytokines in memory B cell differentiation, cytokines play a key role in enhancing the efficacy of resultant memory B cell populations. In primary immune responses, isotype switching from IgM or IgD to IgG, IgE or IgA, supported by IL‐4 [88], IL‐9 [89] and IL‐21 [103] further enhances the specificity of the immune response [110]. Similarly, in humans, IL‐10 receipt by B cells can enhance B cell survival, proliferation, differentiation and isotype switching to IgG4 [99].

The emergence of functionally distinct memory B cell subpopulations can be driven by cytokine signaling. Upon receipt of IL‐2, human and mouse memory B cells can upregulate an immunoregulatory phenotype and secrete IL‐10 to restrain immune activation and restore homeostasis [92, 93, 98]. Conversely, IL‐6 and tumor necrosis factor (TNF)α blockade can dampen memory B cell hyperactivation in rheumatoid arthritis [91, 96]. Furthermore, it is becoming apparent that Type I and Type II interferons can induce the development of functionally unique interferon‐primed memory B cell subpopulations [22, 23, 24, 25, 26, 27].

### 3.2 Precise Regulation of the Timing and Magnitude of Interferon Signaling Is Necessary for Effective Immune Responses

#### 3.2.1 Interferon Signaling in Acute and Chronic Viral Infection

Interferons were first described in 1957, when Type I interferon was identified as an inhibitor of influenza virus replication in vitro [111]. It was later shown that diverse interferons, classified as Type I, II and III interferons, are essential mediators of early viral infection [112]. Interferons are synthesized by immune cells, and in the case of Type I interferon, host cells upon viral invasion [112, 113, 114]. All classes of interferons serve to enhance local antiviral defenses through autocrine and paracrine signaling [112, 113, 114]. The antiviral effects of interferons can be attributed to the induction of over 300 distinct interferon‐stimulated genes (ISGs) [115]. Of these genes, only 51 have been implicated in the direct suppression of viral replication in the local environment: the remainder facilitate broader systemic immune responses through the modulation of both innate and adaptive immune responses [115, 116].

When cytokine responses are dysregulated or fail to resolve, high levels or persistent inflammation can be pathological, driving tissue damage, aberrant immune cell activation and severe disease [117, 118]. In human MERS‐CoV and SARS‐CoV2 patients, there is persistent Type I interferon signaling [119], and a positive correlation between levels of Type I interferon in serum and viral load [120, 121]. In these infections, high ISG expression in peripheral blood mononuclear cells (PBMCs) both during and persisting after the acute phase of infection correlates with severe infection [119, 121, 122]. ISG upregulation in PBMCs is similarly a shared hallmark of human chronic viral infections, including HIV‐1 [123], chronic HCV [124] and CMV [125]. Notably, Type I interferon production and ISG expression in both SARS‐CoV [126] and HIV [80] infection are delayed in vitro. Together, this suggests that interferon production in severe and chronic viral infection is (1) delayed following viral exposure, and (2) sustained over time.

Due to the delay between viral encounter and the appearance of symptoms, it is difficult to assess human interferon induction following viral challenge in vivo. However, there is evidence from mouse models that suggests the in vivo kinetics of early interferon signaling may mirror that observed in vitro [8]. Differences in the dynamics of interferon production in acute and chronic viral infection have been characterized using the murine disease model lymphocytic choriomeningitis virus (LCMV) [8]. In this model, various viral strains can be used to preferentially induce either a resolving (LCMV‐WE or LCMV‐Armstrong) or persistent (LCMV‐Docile or LCMV‐Clone 13) infection, with minimal antigenic variation [127, 128]. In acute infection, interferons are rapidly induced upon pathogen exposure [8, 129]. This response is short‐lived, with interferon signaling resolving prior to the resolution of infection [8, 129]. As observed in human chronic viral infections, in chronic LCMV infection, there is a delayed onset of interferon signaling, that is sustained at a higher level over time [8, 130]. A similar phenotype is observed in SARS‐infected BALB/c mice, where delayed Type I interferon induction relative to viral replication is associated with high viral titers and lethal disease [79]. In this model, administration of Type I interferon early in infection can reduce disease burden and improve viral control [79].

#### 3.2.2 Interferon Signaling in Autoimmunity

There is a clear correlation between the kinetics of interferon‐mediated responses and the efficacy of viral clearance: is this the only context in which dysregulated interferon signaling modulates the immune system? While the early study of interferons focused on their role in viral infection, it is now apparent that high systemic interferon and the failure to resolve interferon‐mediated responses are characteristic of autoimmunity [131]. In patients with systemic autoimmune diseases, including systemic lupus erythematosus (SLE) [132, 133, 134], systemic sclerosis [135, 136], dermatomyositis [137], Sjögren's syndrome [138] and rheumatoid arthritis [139], elevated interferon in serum and ISG upregulation in PBMCs are diagnostically relevant measures. In many cases, this is further associated with disease severity, as seen in viral infection [140, 141].

While a correlative link between dysregulated interferon signaling and disease pathology is well‐recognized, the underlying immune mechanisms are less clear. The ability of interferons to drive both productive and pathogenic immune responses in different contexts complicates the interpretation of ISG signatures in patients [140, 141]. This is evident when studying the effects of interferon on memory B cells. In immune contexts associated with chronic interferon signaling, including chronic viral infection and autoimmunity [76], there is extensive remodeling of the memory B cell compartment [22, 23, 24, 25, 26, 27]. The significance of this remodeling has been an area of controversy, and understandings of the functional capabilities of resulting memory B cell subpopulations have evolved over time.

### 3.3 T‐Bet^+^ Memory B Cells Are Primed by Type II Interferon to Respond to Innate‐Like Stimuli

One memory B cell subpopulation is well‐researched, but inconsistently described in the literature. This subset, referred to as “age‐associated” [142], “exhausted” [22], “tissue‐like” [15], or “atypical” [143] is expanded in malaria, autoimmunity, age‐associated immunodeficiency, chronic viral infection, and some cancers [26, 142]. A common feature of this population is the expression of the transcription factor T‐bet; a key regulator of Type 1 immune responses [22, 26, 144, 145, 146]. Furthermore, these cells often share phenotypic commonalities, including the downregulation of canonical memory B cell markers CD27 (humans) and CD21 (humans and mice) and the upregulation of CD11c (humans and mice) [22, 25, 26, 70, 71]. In this review, they will henceforth be referred to as T‐bet^+^ memory B cells.

**FIGURE 1:**
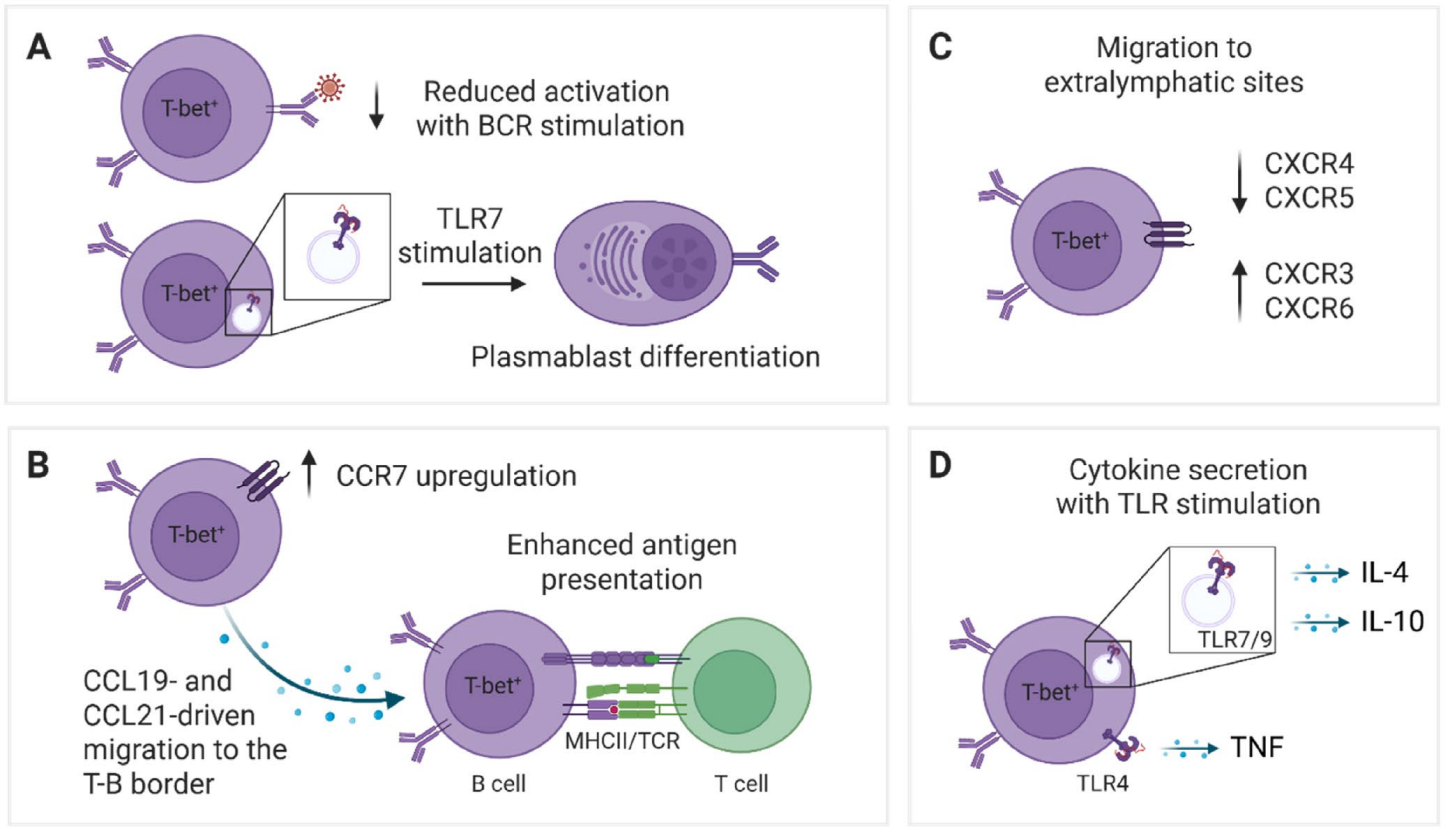
Schematic demonstrating the functions of T‐bet^+^ memory B cells. (A) T‐bet^+^ memory B cells have reduced activation capacity upon BCR stimulation, but differentiate robustly into plasmablasts with TLR7 stimulation. (B) T‐bet^+^ memory B cells upregulate CCR7, and migrate to the T‐B cell border to present antigen to T cells. (C) T‐bet^+^ memory B cells in some contexts downregulate CXCR4 and CXCR5 and upregulate CXCR3 and CXCR6, driving migration to extralymphatic sites. (D) TLR stimulation drives cytokine production by T‐bet^+^ memory B cells.

When T‐bet^+^ memory B cells were initially characterized, they were thought to represent a dysfunctional B cell population, analogous to exhausted T cells [22]. In HIV and CMV, these memory B cells express high levels of inhibitory receptors implicated in the suppression of BCR‐mediated signaling, including CD22, CD72, LAIR1, CD85j, and the IgA‐binding receptor FcRL4 [22, 147, 148, 149]. Furthermore, this memory B cell population shares some characteristics with anergic B cells, including high basal phosphorylation of ERK, elevated cytosolic Ca^2+^ and sustained NFAT activation [150, 151]. Correspondingly, T‐bet^+^ memory B cells are refractory to BCR stimulation once formed, and have reduced capacity to proliferate in vitro [25, 152] (Figure 1A). However, T‐bet^+^ memory B cells are not unique to dysregulated immune environments. Instead, these cells constitute a minor component of the typical immune repertoire in healthy humans [2, 143, 145, 146, 153, 154]. Historically, it was hypothesized that T‐bet^+^ memory B cells may develop from canonical memory B cells experiencing a progressive and hierarchal loss of function due to sustained antigenic stimulation [155]. However, in mice, there is no evidence of this functional loss at the transcriptome level, with T‐bet^+^ memory B cells responding to acute viral infection highly similar transcriptionally to those responding to chronic viral infection [8]. Instead, recent work suggests that T‐bet^+^ memory B cells may represent a specialized memory B cell subset that is uniquely activated independently of BCR stimulation [25, 26].

#### 3.3.1 T‐Bet^+^ Memory B Cells Are Activated Through TLR7 and TLR9

T‐bet^+^ memory B cells upregulate TLRs and respond robustly to TLR7 and TLR9 stimulation [25, 26]. In vitro, stimulation of T‐bet^+^ memory B cells with TLR7/9 can drive proliferation, plasmablast differentiation and antibody secretion [25] (Figure 1A). It has been reported that BCR‐mediated memory B cell activation is reduced in autoimmune patients who have an increased frequency of T‐bet^+^ memory B cells in their immune repertoire [156, 157]. Paradoxically, SLE is characterized by B cell hyperactivity; a phenotype that may be attributed to an increase in responsiveness to nuclear autoantigens through TLR7‐mediated signaling cascades [24, 158]. Similarly, immune contexts associated with expanded T‐bet^+^ memory B cell populations can be associated with hypergammaglobinemia [159, 160, 161]: TLR‐mediated activation of T‐bet^+^ memory B cells by ongoing pathogen burden or damage‐associated molecular patterns may contribute to these dysregulated antibody responses. Together, this demonstrates that T‐bet^+^ memory B cells do retain some canonical memory B cell functions; however, these are initiated independent of BCR stimulation [25, 26].

#### 3.3.2 T‐Bet^+^ Memory B Cell Development Relies on Interferon‐γ

It was once thought that T‐bet^+^ memory B cells were expanded in dysregulated immune contexts because the cells themselves were dysfunctional, and contributed to disease pathogenesis [22]. However, their expansion may instead be explained by the immune signals they receive in dysregulated microenvironments. Chronic viral infection and autoimmunity are associated with chronic BCR stimulation [150], dysregulated TLR signaling [162, 163], and altered interferon dynamics [117, 118, 119]. Correspondingly, in human B cells, T‐bet^+^ memory B cell formation is dependent on synergistic effects of BCR signaling [150], TLR7/9 stimulation [164, 165], and interferon‐γ receipt [94, 95].

In aged and autoimmune mice, T‐bet^+^ memory B cell development is dependent on BTK signaling downstream of the BCR [150]. Furthermore, in human B cells in vitro, stimulation through TLR9 and the interferon‐γ receptor both enhances BTK1 phosphorylation, and drives the upregulation of T‐bet [166]. Ablation of BTK1 signaling in human B cells derived from SLE and multiple sclerosis patients diminishes T‐bet^+^ memory B cell formation [150, 166]. Interferon‐γ has also been shown to directly drive T‐bet expression in influenza and *Heligmosomoides polygyrus* infections [95]. Autocrine interferon‐γ signaling can similarly promote the expression of genes characteristic of T‐bet^+^ memory B cells in SLE [94]. Together, this suggests that the combination of antigen, TLR agonists and interferon in the microenvironment can drive T‐bet expression and T‐bet^+^ memory B cell development. This may occur, in part, due to alterations in BCR signaling strength, with an additional role for chronic interferon‐γ in directly driving excessive T‐bet expression.

#### 3.3.3 T‐Bet^+^ Memory B Cells Are Not Functionally Impaired

Studies show that T‐bet^+^ memory B cells are functional in viral infection; however, in some cases, these functions differ from the canonical memory B cell function of antibody‐secreting cell production. Some studies have suggested that T‐bet^+^ memory B cells may be specialized for antigen presentation [167, 168]. In aged mice, T‐bet^+^ memory B cells upregulate CCR7 and migrate to the border of the T and B cell zones in the spleen in response to CCL19 and CCL21 [167] (Figure 1B). In this niche, T‐bet^+^ memory B cells present antigen with high efficacy, promoting sustained T cell‐mediated responses through stable interactions with CD4^+^ T cells [167]. It has also been suggested that this antigen presentation may help to sustain germinal center reactions with ongoing antigen burden [168]. However, whether persistent germinal centers contribute to productive immune responses in dysregulated immune landscapes is under debate [169, 170]. Thus, it is unclear whether this is a beneficial immune outcome.

By contrast, in HIV and CMV, migration of T‐bet^+^ memory B cells to the spleen and lymph nodes is reduced, due to CXCR4 and CXCR5 downregulation [147, 171, 172]. Instead, in HIV, CXCR3 and CXCR6 are upregulated, driving migration to extralymphatic sites and promoting the enrichment of T‐bet^+^ memory B cells in peripheral tissues [148, 172, 173] (Figure 1C). It may be that there are functionally distinct roles for T‐bet^+^ memory B cells depending on their localization and the local signals they encounter in distinct immune niches. Finally, T‐bet^+^ memory B cells secrete cytokines in response to TLR stimulation, including IL‐10 and IL‐4 in response to TLR7/9 [25], and TNF in response to TLR4 [174] (Figure 1D). The in vivo significance of T‐bet^+^ memory B cell cytokine production is unclear: in splenic T‐bet^+^ memory B cells, they may be involved in paracrine signaling to neighboring T cells. Alternatively, they may contribute to the systemic proinflammatory environment in dysregulated immune contexts.

In acute infection and repeat vaccination, T‐bet^+^ memory B cells can contribute to productive antibody responses [154, 175, 176]. A T‐bet^+^ population co‐expressing the IgG‐binding regulatory receptor FcRL5 [149] has been described as effector‐like memory B cells in humans [175] and mice [177]. FcRL5^+^Tbet^+^ memory B cells are poised, by both metabolic and epigenomic mechanisms, for the production of high‐affinity neutralizing antibodies [176]. Correspondingly, in vitro, T‐bet^+^ memory B cells can rapidly differentiate to form antibody‐secreting cells [154, 175]. Elevated FcRL5 expression is associated with affinity maturation [154], with higher levels of FcRL5 expression associated with greater accumulation of somatic mutations [178]. Furthermore, these memory B cell populations are class‐switched, further enhancing the specificity of secondary immune responses they induce [178]. In vivo, loss of T‐bet^+^ memory B cells ablates the IgG2a antibody responses in murine antiviral recall responses, suggesting that these memory B cells may play a productive role in viral clearance [153]. Similarly, in human tonsils, IgD^+^ memory B cells transcriptionally resembling T‐bet^+^ memory B cells localize to mucosal surfaces, where they differentiate into antibody‐secreting cells [63]. This is thought to drive rapid humoral immune responses against inhaled pathogens [63].

Collectively, it appears that T‐bet^+^ memory B cells are not functionally impaired and should instead be considered a canonical memory B cell population adapted to respond to innate‐like stimuli. What is unclear is the implications of this population being expanded in altered immune contexts. Is this a population specifically induced to enhance immunity in inflammatory environments, or instead, is its emergence an adaptation to an unfavorable immune landscape? Accordingly, do they play a productive role or exacerbate inflammation to drive pathogenesis? To answer this question, further work is needed to delineate the effects of T‐bet^+^ memory B cell expansion from those of CD21^+^ memory B cell loss observed in the same contexts. Understanding this may allow for identification of context‐specific roles for T‐bet^+^ memory B cells. Furthermore, it may be that there is further unexplored heterogeneity within this subset that can drive divergent immune responses in different immune responses.

### 3.4 Type I Interferon Drives the Emergence of a Distinct Memory B Cell Subpopulation With Unknown Functional Capabilities

There is a population of memory B cells that similarly emerges in pro‐inflammatory immune environments, but does not express T‐bet. Instead, this population broadly upregulates ISGs and is primed by Type I interferon signaling. In our work, which used single cell RNA and ATAC sequencing to characterize the differences in memory B cell responses in murine acute and chronic viral infection, this population was expanded in the chronic infection model LCMV‐Docile [8]. Gene set enrichment analysis revealed an enrichment for IFN‐α and IFN‐γ response hallmark gene sets within this population compared to other memory B cell subsets. Notably, the formation of Type I interferon‐primed memory B cells was reduced with both systemic IFNAR blockade and ablation of B cell‐intrinsic IFNAR signaling. This suggests that the emergence of this population is governed by receipt of Type I interferon by B cells. A population of T‐bet^+^ memory B cells was also observed in our dataset, but this subset was distinct to the Type I interferon‐primed subset.

The characterization of human lymphocyte populations in a range of pro‐inflammatory immune contexts has uncovered that a highly similar interferon signature is conserved across a range of pathologies associated with persistent Type I interferon signaling [18, 179, 180, 181, 182]. Peripheral blood interferon gene signatures are characteristic of the immune landscape in Sjogren's syndrome [179], persistent inflammatory rheumatoid arthritis [180], tuberculosis [181], diabetes [181] and fibromyalgia [182]. A specific B cell signature has also been identified in SLE [18]. While studies into these conditions have not specifically focused on memory B cell responses, it is reasonable to predict that similar ISG‐expressing memory B cell populations may be part of the immune repertoire of these patients. Further evidence supporting the existence of Type I interferon‐associated memory B cells in humans can be found in single cell analysis of human memory B cells in the tonsils, where a subset of memory B cells has enhanced interferon‐related gene activity [59]. But how do Type I interferon‐primed memory B cells contribute to the immune repertoire?

The study of altered memory B cell subpopulations in human disease is limited by their low frequency in blood and the limited techniques to study memory B cell function ex vivo. In mouse‐derived B cells, Type I interferon‐primed memory B cells have reduced proliferative and differentiation capacity in vitro upon stimulation with CD40L, anti‐Ig and IL‐21 [8]. However, there is insufficient evidence to decisively conclude what their functional capacity is in vivo. In human models, the role of Type I interferon‐primed memory B cells has not been studied: their functional capabilities can only be speculated to be by cross‐referencing known characteristics of the total B cell pool with animal models and memory B cell‐specific phenotypic characteristics. In a mouse model of severe SLE, TLR7 can drive induction of B cell intrinsic Type I interferon production by transitional B cells [183]. In the absence of this production, and specifically interferon‐β signaling, the transcriptomic landscape of the B cells is transformed, with a reduction in interferon‐α production and decreased *Tlr7* expression [183]. Similarly, in human SLE patients, upregulation of TLR7 and TLR9 in PBMCs correlates with enhanced levels of interferon‐α [163]. Together, this may suggest that B cell pathogenesis in SLE can, in part, be driven by positive feedback loops between Type I interferons and TLR7. Memory B cells in SLE likewise upregulate TLR7, and exhibit enhanced responsiveness upon stimulation [158]. By this logic, memory B cell production of Type I interferon and autocrine signaling may be implicated in the establishment and maintenance of a Type I interferon signature in memory B cells.

### 3.5 How Are Memory B Cells Primed by Different Signals in the Same Inflammatory Environment?

The existence of two distinct interferon‐associated memory B cell subpopulations, primed by either Type I or Type II interferon, raises interesting questions about how the microenvironment can influence memory B cell identity. In chronic LCMV infection, both Type I and Type II interferon‐primed memory B cells develop within the same systemic proinflammatory environment, and yet, they undertake different development trajectories [8]. The intracellular events following cytokine receipt by B cells are generally poorly defined, making it difficult to speculate as to how B cells may regulate and synthesize environmental signals in cases of co‐receipt. The dissection of these signals is further complicated by the overlap in detectable gene signatures and surface marker expression driven by Type I and Type II interferons [184].

In SLE, there is evidence of a T‐bet‐expressing memory B cell population that may develop from the co‐receipt of interferon‐γ and interferon‐β. In SLE, T‐bet expression can drive the upregulation of CXCR3 to promote B cell infiltration at sites of inflammation [185]. Conversely, receipt of Type I interferons downregulates CXCR5 expression [185]. Functionally, this altered chemokine receptor expression profile reduces B cell migration in response to CXCL13, reducing T‐B cell colocalization [185]. The expression of CXCR3 on memory B cells in SLE may indicate that this subset is primed by both Type I and Type II interferons [185]. Notably, CXCR5^−^CXCR3^−^ B cells are also expanded in SLE independently of the CXCR5^−^CXCR3^+^ subset, which may imply that not all memory B cells primed by Type I interferon also respond to Type II interferon [185]. Reduced CXCR5 and increased CXCR3 expression in B cells is also observed in rheumatoid arthritis patients [186], suggesting that this is not an SLE‐specific phenomenon. It may be that different memory B cell subsets have different capacities to respond to systemic interferon: in which case, what are the molecular drivers underlying this differential responsiveness? Alternatively, these different memory B cell subsets may emerge due to differences in localization. The upregulation of CCR7 in T‐bet^+^ memory B cells and subsequent interactions with T cells at the T‐B zone border may drive the emergence or sustainment of their phenotype [167]. However, it is unclear whether these cells develop within this niche or whether they develop elsewhere and migrate to fulfill their function as antigen presenting cells. A role for spatial organization within secondary lymphoid organs in modulating B cell activation and differentiation has not been comprehensively described.

## 4 The Specific Niche of the Tumor Microenvironment Drives the Emergence and Expansion of Memory B Cell Subsets

In antitumor immunity, the immune repertoire is comprised of populations primed in both secondary lymphoid organs and in the unique tumor microenvironment [187, 188, 189]. Can the microenvironmental differences between the periphery and the tumor microenvironment subsequently drive the emergence of different B cell populations? The tumor microenvironment is comprised of an intricate network of cellular and acellular components, including fibroblasts, endothelial cells, immune cells, the extracellular matrix and vasculature tissue (reviewed elsewhere [188, 189]). In the tumor microenvironment, immune cell function is mediated by cell–cell interactions and cancer‐intrinsic and ‐extrinsic paracrine signaling [189, 190]. Reciprocally, immune cell cytokine secretion can promote tumor progression or regression, depending on their phenotype [191].

Immune‐infiltrating, or “hot” tumors, are generally associated with higher patient responsiveness to anticancer immunotherapy and better prognostic outcomes [192]. The secretion of cytokines into the microenvironment by infiltrating immune populations drives tumor regression [191]. However, the specific nature of the cytokines produced, cytokine concentration, and the duration of cytokine signaling can shift the balance within the tumor microenvironment from productive to harmful [193]. Cytokines within the tumor microenvironment can promote immune cell function, driving antitumor responses [191]. However, the failure to resolve acute inflammation and disruption of homeostasis can promote cancer formation by driving cell proliferation, promoting tumor cell survival, and facilitating angiogenesis [194]. Furthermore, regulatory immune populations, including regulatory T and B cells, can produce immunosuppressive cytokines, dampening the function of antitumor immune populations and facilitating tumor cell escape [195]. Chemokines can similarly promote immune cell infiltration into the tumor microenvironment and drive tumor growth, survival, and metastasis [196].

The broad targets and multifaceted functions of interferons mean that their function in the tumor microenvironment is likewise highly context‐dependent. There is significant overlap between the roles of Type I and Type II interferons in systemic immune responses and their antitumor functions in the tumor microenvironment, including enhanced activation of several innate populations, enhanced differentiation of adaptive immune populations and decreased function of regulatory populations (reviewed elsewhere [197, 198, 199]). In tumor B cells, Type I interferon enhances B cell activation [197], while Type II interferon promotes B cell proliferation following primary antigen exposure and drives IgG2 antibody class switching [200]. However, sustained interferon signaling can drive the dysfunction of dendritic cells [193, 201] and CD4^+^ T cells [201], and induce apoptosis of CD8^+^ T cells [202]. Similarly, prolonged and excessive Type II interferon signaling can stimulate the regulatory effector functions of regulatory T cells [203] and myeloid derived suppressor cells [204], indirectly dampening the antitumor action of other local immune populations.

Memory B cell infiltration is a critical component of anticancer immunity, that frequently correlates with favorable prognostic outcomes for patients [16]. In tumors, memory B cells are often characterized by CD27 expression, and the lack of other B cell subset markers [205, 206, 207], though CD27^−^ memory B cell populations have also been recognized [208]. However, B cell remodeling within the tumor microenvironment can be counterproductive and support pro‐cancer immune responses that drive cancer growth and metastasis [209]. To understand this dichotomy, it is critical to understand the drivers of memory B cell identity in the tumor microenvironment.

### 4.1 Memory B Cell Phenotype in Antitumor Immunity Is Influenced by Interferon‐Driven Tertiary Lymphoid Structure Formation, and Is Linked to Prognostic Outcome

#### 4.1.1 Tertiary Lymphoid Structure Formation

In the tumor microenvironment, B cells can be assembled into tertiary lymphoid structures: organized aggregates of lymphocytes within the tumor microenvironment [210, 211, 212]. Maturation of tertiary lymphoid structures in tumors can drive ectopic germinal center formation [210], and thus presumably, germinal center‐independent and dependent memory B cell subsets can emerge over time. Spatial transcriptomic approaches have recently identified interferon as a molecular driver of tertiary lymphoid structure formation in nasopharyngeal carcinoma [213]. In this model, interferon promotes CXCL9 production in high endothelial venules, driving T cell recruitment [213]. While direct evidence of this mechanism has not been observed in other cancers, there are some indirect indications that interferon might be similarly important in mouse models of breast cancer [214]. In this model, STING activation through interferon signaling synergizes with lymphotoxin (LT)βR signaling. This signaling is associated with tertiary lymphoid structure formation, immune cell fitness, and long‐term survival [214]. Both of these mechanisms driving tertiary lymphoid structure formation in tumors are T cell‐driven: the environmental factors acting on B cells in this context are not well‐defined.

This mechanism is similar to that observed in tertiary lymphoid structure formation in other contexts. In the lungs, both the initial formation of tertiary lymphoid structures and ectopic germinal center formation are reliant on Type I interferon signaling [215, 216]. Type I interferon induces LTα production, stimulating stromal cells to produce CXCL13 and drive CXCL5‐mediated B cell recruitment [215]. Type I interferon also directly and indirectly stimulates stromal cells to secrete the T cell attracting chemokines CXCL9, CXCL10, CCL19 and CCL21, driving immune cell aggregation and facilitating T‐B cell interactions [215]. Whether similar signals also act on B and T cells in the tumor microenvironment has not been explored.

#### 4.1.2 Correlating Tertiary Lymphoid Structure Formation With Clinical Outcomes

**FIGURE 2:**
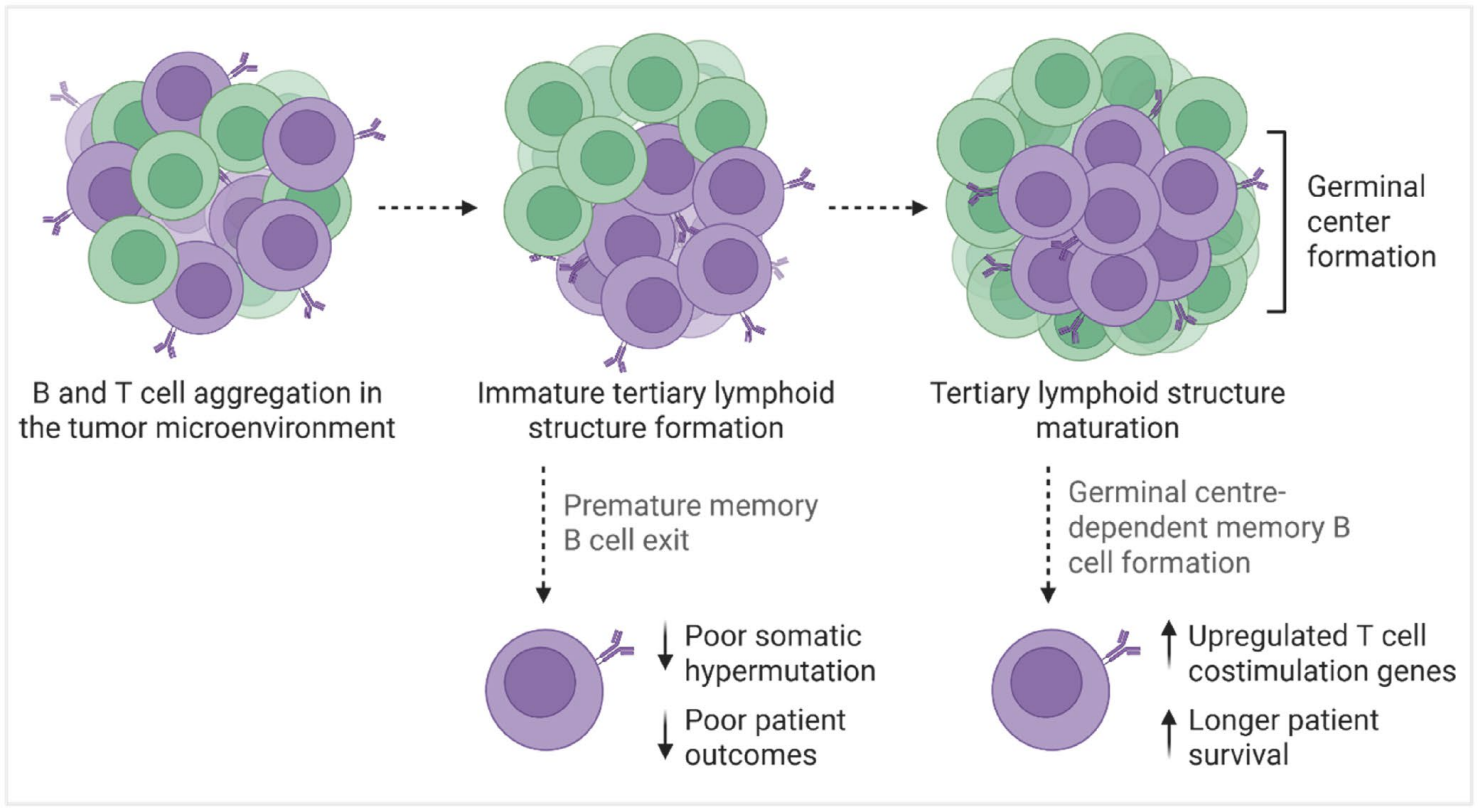
Simplified schematic of tertiary lymphoid structure aggregation. Tertiary lymphoid structure maturation and germinal center formation is associated with patient survival. Premature B cell exit from immature tertiary lymphoid structures correlates with poor patient outcomes. Purple cells are B cells; green cells are T cells.

Germinal center formation within tertiary lymphoid structures and antibody‐secreting cells in the tumor microenvironment are associated with strong anti‐tumor T cell responses and good responsiveness to immunotherapy [207, 211, 212, 217]. Specifically, a subset of FcRL4^+^ tumor‐associated memory B cells expressing high levels of T cell coactivation genes can be predictive of longer survival [206]. By contrast, a separate population of FcRL4^+^ memory B cells with poor somatic hypermutation has been shown to correlate with poor responses to therapeutic intervention, disease progression, and poor patient outcomes [218]. These populations are found in immature tertiary lymphoid structures and accumulate outside of germinal centers [218] (Figure 2). The drivers of these populations are not fully elucidated; however, their development appears to be dependent on the receipt of both tumor‐derived and tumor‐independent signals. Several tumor‐associated memory B cell subpopulations are highly homologous to circulating populations found in chronic viral infection and autoimmunity, suggesting that they may share microenvironmental signals and molecular drivers.

### 4.2 Phenotypically Similar but Functionally Distinct Memory B Cell Populations Are Predictive of Patient Survival and Response to Therapeutic Intervention

In healthy patients, FcRL4^+^ memory B cells are a minor constituent of the memory B cell pool, first identified in human tonsils [219] and later described in mucosal associated lymphoid tissues, where they mediate responses to commensal microbes [63, 220]. This population is greatly expanded in the tumor microenvironment; particularly in poor prognosis tumors [71, 209]. Examining the transcriptome, phenotype, and functional capacity of tumor‐derived FcRL4^+^ memory B cells from across a range of pathologies reveals that this population shares some striking similarities with the T‐bet^+^ memory B cell population enriched in chronic viral infection and autoimmunity. Tumor‐resident FcRL4^+^ memory B cells downregulate CD27 and CD21, upregulate CD11c, and express ZEB2 and T‐bet [71, 218]. Correspondingly, T‐bet^+^ memory B cells in HIV, CMV, and rheumatoid arthritis can also upregulate FcRL4 [221, 222, 223].

#### 4.2.1 Two FcRL4 ^+^ Memory B Cell Subsets Are Associated With Poor Prognosis

FcRL4^+^ tumor‐associated memory B cells have been further subdivided into multiple functionally distinct subclasses. Two single‐cell pan‐cancer screens performed by Ma et al. and Yang et al. demonstrate the extent of FcRL4^+^ memory B cell diversity [206, 218]. In these screens, a subset of FcRL4^+^ memory B cells was associated with poor prognosis. These memory B cells displayed evidence of inflammation‐induced stress responses, including the upregulation of heat shock genes and reduced responsiveness to BCR stimulation [206, 218]. This subset could be further subdivided into checkpoint‐positive and negative populations, according to their expression of suppressive checkpoint molecules including PD‐1, CD274, CTLA4, CD39, LAG3, and TIM‐3 [206, 218]. This correlates with differences in interactions with tumor‐resident T cells: checkpoint‐negative memory B cells fail to interact with CD4^+^ T cells, while checkpoint‐positive memory B cells can suppress T cell function [218]. When an analogous population to the latter was isolated from head and neck squamous cancer tumors and stimulated with antigen in vitro, these cells failed to differentiate into antibody‐secreting cells [71]; however, the functional capabilities of this subset in response to alternative forms of activation have not been explored. Similarly, tumor‐derived non‐switched Tbet^+^CD11c^+^ memory B cells from hepatocellular carcinoma patients adopted a regulatory‐like phenotype, expressing high levels of PD‐L1, CD26 and granzyme B [224]. This population can subsequently suppress peripheral blood B cells and sequentially dampen peripheral T cell co‐stimulation [224].

The factors that drive the emergence of these two subsets are also not well understood, though it may be related to their origin source. Ma et al. suggest that the checkpoint‐expressing memory B cell population has an extrafollicular origin and may arise from naïve B cells induced by interferons [218]. Correspondingly, in head and neck squamous cancer, TLR7 activation, Type II interferon receipt and IL‐21 can drive the accumulation of extrafollicular‐derived, checkpoint‐expressing memory B cells [71]. Comparatively, Yang et al. could not definitively exclude the possibility of checkpoint‐negative memory B cells originating from the germinal center [206]. Thus, these two populations may emerge from divergent developmental pathways.

#### 4.2.2 A Proportion of FcRL4 ^+^ Memory B Cells Are Linked to Favorable Prognoses

A subset of FcRL4^+^ memory B cells, defined by Yang et al. as tumor‐associated atypical B cells, may have anti‐tumor properties [206]. This population has an activated phenotype, defined by high expression of *CD80*, *CD86*, and *FAS* (CD90), and the upregulation of cytokine and chemokine receptors including *IFNGR1*, *CXCR3*, and *CCR1*. Notably, this population upregulates genes associated with both antigen processing presentation via MHC Class II and costimulatory ligand‐receptor pairs involved in CD4^+^ T cell co‐stimulation. Furthermore, these cells spatially reside in close proximity to CD4^+^ T cells. Together, this suggests that a population of FcRL4^+^ memory B cells may enhance antitumor immunity through efficacious antigen presentation. A similar population has been identified in nasopharyngeal carcinoma, in which a population of CD20^+^FcRL4^+^ B cells upregulates pathways associated with antigen presentation and cell adhesion while downregulating antibody response pathways [225]. Instead of being specialized for CD4^+^ T cell activation, this population has the capacity to cross‐present tumor antigens to CD8^+^ T cells via MHC Class I; a process facilitated by Type II interferon receipt [225]. Given that tumor‐associated atypical B cells were not proximally located to CD8^+^ T cells, these populations may be indicative of further diversification of the FcRL4^+^ memory B cell pool in the tumor microenvironment.

What is unclear is how these divergent FcRL4^+^T‐bet^+^ memory B cell populations develop, and how functionally distinct FcRL4^+^ populations are related to one another. Do antitumor FcRL4^+^ memory B cells gradually lose their functionality with prolonged antigen exposure? Alternatively, can protumor FcRL4^+^ subpopulations recover functionality under the right conditions in the tumor microenvironment? Trajectory analysis and quantification of the degree of BCR sharing between memory B cell clusters suggests that neither may be the case: instead, tumor‐associated atypical B cells seem to be the terminal state among memory B cell subsets, as the last subset to emerge in pseudo time [206]. The authors hence suggest that this subset may emerge from classical memory B cells that later adopt an interferon‐associated gene signature [206]. It may be that there are instead undefined environmental drivers causing the emergence of distinct populations, or a role for cell‐intrinsic differences in memory B cell precursor populations. Given the role of FcRL4 as a dampener of BCR signaling [226], antigen affinity and BCR signaling strength may be implicated in the difference in responsiveness between different FcRL4^+^ subpopulations.

### 4.3 Memory B Cell Responses to Type I Interferon May Enhance Antitumor Immunity

Given that the presence and persistence of Type I interferon signaling can drive the emergence of altered memory B cell subpopulations in interferonopathies, and the kinetics of interferon signaling within the tumor microenvironment can correlate with different immune outcomes, does Type I interferon likewise play a role in antitumor memory B cell responses? There is evidence to suggest this is the case. Integration of single cell sequencing datasets from seven cancer types revealed a population of memory B cells that upregulated ISGs but did not have detectable T‐bet expression [227]. This may represent a Type I interferon‐primed memory B cell population that can form naturally in several tumor types. Furthermore, single‐cell sequencing of human tumor infiltrating lymphocytes in breast, colorectal, ovarian, and lung cancers uncovered a population of B cells expressing the interferon‐associated B cell checkpoint molecule TIM‐1 [228, 229]. TIM‐1 is a promising target for tumor immunotherapy that is upregulated on B cells to dampen B cell activation [198]. Specifically, TIM‐1^+^ B cells are enriched in treatment‐naïve patients and downregulated with checkpoint blockade [228]. Transcriptomic analysis of treatment‐responsive patients versus treatment naïve correspondingly revealed augmented Type I interferon, B cell activation, antigen processing, antigen presentation, and T cell co‐stimulation signatures [228].

In mouse models, co‐administration of an anti‐TIM‐1 antibody with a STING agonist to induce Type I ISGs could remodel the tumor microenvironment in an anti‐PD1‐resistant HCA‐1 mouse model, enhancing B cell responsiveness and augmenting CD8^+^ T cell‐mediated antitumor responses [228]. This provides evidence that, in both mice and human patients, B cell responsiveness to Type I interferon may play a key role in mediating antitumor immune responses, and targeting hyporesponsive B cell populations may be a viable therapeutic strategy for patients resistant to immune checkpoint blockade. While these studies did not discriminate between naïve and memory B cells expressing TIM‐1, there is some evidence in other disease contexts that antigen‐experienced CD27^+^TIM‐1^+^ memory B cells do have regulatory capabilities [230]. This suggests that TIM‐1^+^ memory B cells may be of clinical relevance in the tumor microenvironment.

Further research into tumor‐associated memory B cell subpopulations to determine the molecular drivers underlying their formation is critical. By understanding how memory B cells can be remodeled within the tumor microenvironment, their viability as targets for anticancer therapies can be assessed. By defining these determinants of memory B cell identity in tumors, we can also more comprehensively define non‐canonical memory B cell subsets and look to identify similar populations in the circulating memory B cell pool. This may help resolve the discordance in the functional capabilities of T‐bet^+^ populations across disease contexts.

## 5 Molecular Regulation of Memory B Cells: Remodeling Memory B Cell Identity for Therapeutic Benefit

It is evident that the microenvironment plays a key role in memory B cell functionality, and in driving the emergence of distinct subpopulations within the memory B cell pool. This raises several questions: can the memory B cell response be remodeled through modulation of the microenvironment? In aberrant immune contexts, is it possible to shift the representation of memory B cell subpopulations within the total pool through therapeutic intervention, and will this promote better clinical outcomes? By inducing microenvironmental changes, can certain memory B cell subsets be preferentially induced in vaccination for better protection?

To answer these questions, it is important to understand how memory B cells are programmed. Memory B cell formation is governed by the cooperative efforts of transcription factors and epigenetic modifiers that expose or inhibit transcription factor binding sites [231]. In B cells, a variety of epigenetic regulators, including histone modifiers [10], DNA demethylases [232] and non‐coding RNA [233, 234], modulate the expansion and contraction of chromatin and regulate gene accessibility. Several recent studies have effectively demonstrated that functionally distinct memory B cell subpopulations are associated with unique transcription factors and have divergent chromatin landscapes [235]. These differences underlie the phenotypic and functional specificity of memory B cells [235]. By examining how memory B cells are regulated and how chromatin can be remodeled during the development of both canonical and newly emerging memory B cell populations, insight can be gained into (1) the timeframes in which memory B cell identity may be determined, (2) the contribution of environmental stimuli to B cell chromatin remodeling, and (3) how distinct memory B cell subsets can emerge from different microenvironmental niches. Understanding these core concepts will reveal how viable memory B cell remodeling is as a therapeutic strategy to target memory B cell functionality.

### 5.1 Epigenetic Remodeling Underlies B Cell Function

When naïve B cells are compared to antigen‐experienced memory B cells, memory B cells have more regions of open chromatin in the memory pool, with greater accessibility of gene loci associated with plasma cell differentiation (*Prdm1* and *Irf4*) and germinal center formation (*Aicda*) [236]. These changes can be mapped back to the dynamic remodeling of the chromatin landscape that occurs with B cell activation [235]. Following antigen encounter, naïve B cells progressively demethylate transcription factor binding sites [235]. These changes in the methylome are epigenetically stable, and are subsequently inherited by downstream memory B cells and terminally differentiated plasma cells [235]. Correspondingly, memory B cells can upregulate primed transcription factors more rapidly and robustly than their naïve counterparts in vitro [236], correlating with faster and more robust secondary germinal center and antibody‐secreting cell responses in vivo [72]. Furthermore, memory B cells have altered expression of genes associated with cell cycle progression [237], signal transduction [238, 239], chemotactic receptors [240, 241], repression of apoptosis [238] and T‐B cell interactions [57], associated with increased longevity and heightened sensitivity to stimulation upon activation [57, 236].

There is evidence that the germinal center reactions drive remodeling of the chromatin architecture, with germinal center‐derived memory B cells exhibiting increased capacity for proliferation, GPCR‐mediated signaling and cytokine responsiveness compared to those of extrafollicular origin [4]. Work by us and others has shown that deletion of epigenetic modifiers associated with germinal center formation and kinetics in mice, including MOZ [242], HELLS [243] and EZH2 [244] can promote premature upregulation of memory B cell markers, or the expansion of memory B cell phenotypes more commonly associated with the extrafollicular pathway. In this way, distinct subpopulations of memory B cells arise according to their origin. In mice, extrafollicular memory B cells more closely resemble naïve follicular B cells, while germinal center‐derived memory B cells share epigenetic features with their germinal center B cell precursors [69]. This correlates with functional differences in memory B cell populations [69]. For example, in vitro and in vivo, germinal center‐derived CD80^+^PD‐L2^+^ have decreased proliferative capacity, but generate more plasmablasts on a per‐cell basis than phenotypically similar cells emerging from extrafollicular reactions [69].

#### 5.1.1 Epigenetic Dysregulation on the X Chromosome May Explain Altered Functionality of SLE B Cells

**FIGURE 3:**
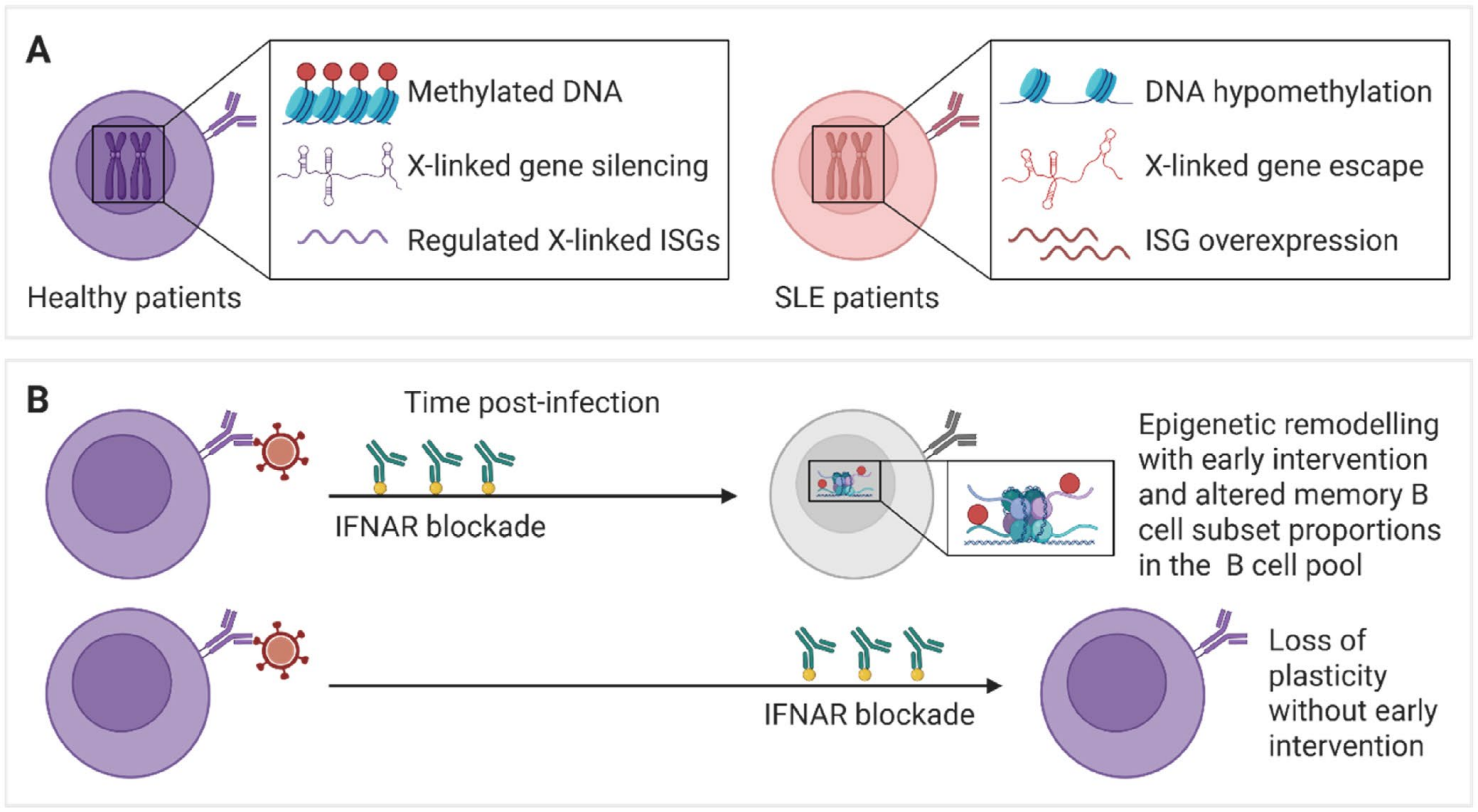
Memory B cell epigenetic remodeling in (A) SLE (B) murine chronic viral infection. (A) SLE is associated with DNA hypomethylation, driving aberrant gene activation. XIST mutations drives X‐linked gene escape, resulting in the overexpression of X‐linked interferon stimulated genes. (B) In LCMV‐Docile infection, early IFNAR blockade can remodel the B cell epigenome and alter the composition of the memory B cell pool. However, B cells are not refractive to remodeling with late intervention.

Some characteristic features of the B cell response in SLE may be explained by epigenetic dysregulation on the X chromosome [245, 246] (Figure 3A). X chromosome inactivation is a unique epigenetic modification, involving heterochromatic reorganization of one X chromosome to silence X‐linked gene expression and prevent a dose‐effect in females [245, 246]. SLE has been linked to alterations in the non‐coding RNA XIST on the inactive X chromosome across all B cell subsets, resulting in a failure to maintain X‐linked inactivation and the escape of X‐linked genes [246]. This impairs dose compensation and drives abnormally high gene expression of X‐linked interferon‐associated genes, including *Tlr7*, the ISG and TLR7 interaction partner *CXorf21*, and proinflammatory regulator *IRAK1* [245]. While expression of these genes has not been specifically quantified in memory B cells, epigenetic dysregulation on the inactive X chromosome may explain B cell hyperresponsiveness to innate‐like stimulation through TLR7 [247]. This would exacerbate TLR7‐mediated interferon production and associated signaling cascades to further upregulate ISG signatures in memory B cells.

### 5.2 The Characteristic Features and Functions of Memory B Cells Are Epigenetically Imprinted Early in Their Developmental Trajectory

It is clear that the epigenetic landscape of a B cell underpins its function, but how are these distinct epigenetic states established? The role of the microenvironment in driving epigenetic reprogramming can be studied by examining memory B cell populations activated by distinct stimuli. When the epigenetic changes induced upon B cell activation in T‐dependent and T‐independent manners are examined, there are clear differences in chromatin landscape that contribute to the divergent trajectories of these populations [235]. In vitro, human B cells stimulated with T‐dependent or T‐independent stimuli have distinct methylation profiles, with increased sites of CpG hypermethylation post‐T‐independent stimulation [235], indicative of higher levels of gene repression [248]. Correspondingly, T‐independent memory B cells functionally lack the sensitivity and extended lifespan of T‐dependent memory B cells [235]. This finding potentially implicates receipt of microenvironmental signals early in B cell activation, including T cell‐derived signals, PRR‐mediated pathogen recognition and the local cytokine landscape in the remodeling of the B cell epigenome.

From this, it is evident that distinct epigenetic signatures can be established from as early as B cell activation and are conserved post‐memory B cell differentiation to drive functional outcomes. Thus, extrinsic signals encountered early in a memory B cell's developmental trajectory can play a critical role in determining its identity.

#### 5.2.1 Memory B Cell Remodeling Requires Early Intervention or Secondary Antigen Encounter

If early signals encountered by B cells can be deterministic of memory B cell identity, does this provide a “window” in which clinical intervention could remodel the memory B cell pool? Our work suggests that this may be possible. In LCMV‐Docile‐infected mice, blockade of Type I interferon signaling can remodel the memory B cell epigenome and alter the relative proportions of memory B cell subpopulations within the total B cell response (Figure 3B) [8]. However, memory B cells were not responsive to manipulation of Type I interferon signaling late in infection, after the formation of the bulk of the memory B cell milieu [8]. This suggests that, while memory B cells have some plasticity during their development, the longer the timeframe post‐infection, the less responsive memory B cells become to intervention.

There is evidence that upon reactivation, memory B cells can again undergo reprogramming. Memory B cells formed in SARS‐CoV‐2 infection are able to re‐differentiate into phenotypically different memory B cell subsets upon vaccination [70]. In this study, individual B cell clones were tracked by B cell receptor signaling, demonstrating that the change in SARS‐CoV‐2 memory B cell phenotype at the population level was attributed to remodeling pre‐existing memory B cells, and not driven by de novo memory B cell generation [70]. Future studies may be needed to examine whether this reprogramming is evident at the epigenomic level, whether it occurs post‐secondary infection, and how it is influenced by the microenvironment. Taken together with our murine studies, this suggests that B cell identity can be manipulated within discrete windows of time post‐antigen exposure, whether this is in primary or secondary response. Therefore, therapeutic intervention may be able to shape the memory B cell compartment, but timing will be critical for success. Furthermore, if memory B cell plasticity can be induced through immunization, it may be possible to tailor vaccinations to selectively induce specific memory B cell subsets.

### 5.3 Local Environmental Cues Are Implicated in the Determination of Memory B Cell Identity

The importance of early priming in the determination of memory B cells is clear; yet, this does not explain how different memory B cell subsets can arise from within a single infectious context where, presumably, they are receiving the same systemic environmental cues. This phenomenon may be explained by differences in cell localization. Distinct memory B cell subsets or their precursors may occupy different niches, resulting in different cell–cell interactions and paracrine signals that drive divergent epigenetic signatures. Autocrine signaling may also contribute to these distinct responses.

Preliminary studies of tissue‐resident memory B cell transcriptional identity provide insight into how spatial isolation of memory B cell populations can drive the emergence of different memory B cell populations. Unlike traditional memory B cell populations, tissue‐resident populations do not recirculate and are instead maintained at sites of interest [2]. Transcriptomic analysis of human and mouse lung‐resident memory B cells demonstrates that they are distinct from circulating counterparts: a phenotype which may be attributed to differences in environmental stimuli [2, 249, 250]. The presence of different structural and immune populations within distinct organs would greatly affect the cell–cell interactions and paracrine signals received by tissue‐specific populations. This may occur, in part, during local naïve B cell activation [2]: in influenza infection, there is an elevated frequency of cross‐reactive memory B cells in the lungs compared to circulating lymphoid organs, driven by persistent germinal center responses within the lungs themselves [251]. However, the bulk of tissue resident memory B cells in the lungs originate from the mediastinal lymph nodes and the spleen, with only a small proportion produced from local activation of naïve B cells in the iBALT [252]. This implies that B cells activated in secondary lymphoid organs can be remodeled following entry into the lungs. While the specific signals driving this remodeling have not yet been defined, how tissue‐specific epigenomic signatures are established, and the functional significance of these signatures, are not yet known.

It is clear that location can play a role in memory B cell identity. There is particular relevance of these findings to tumor‐resident memory B cells, that are spatially isolated from circulating populations and receive unique microenvironmental cues. The impact of the tumor microenvironment on the phenotype and transcriptional landscape of memory B cells has been appreciated, but to what extent memory B cells can be molecularly modified within this environment, and by what extrinsic cues, is an ongoing area of investigation.

### 5.4 B Cell Function, the Epigenome and the Disease‐State Microenvironment Can Be Modulated by Metabolic Programming

As B cells progress through different stages of development, there are dynamic changes in the metabolic pathways they use [253, 254]. As a quiescent population, memory B cells have low energy requirements compared to rapidly proliferating germinal center B cells and antibody‐secreting populations [254]. Accordingly, metabolic changes in the germinal center reactions are important for germinal center exit, with low mTORC1 activity in germinal center B cells associated with the memory B cell fate [44]. Furthermore, the maintenance and long‐term persistence of memory B cells in circulation is dependent on basal levels of mitochondrial autophagy to maintain mitochondrial homeostasis [255]. The reactivation capacity of memory B cells may be, in part, driven by metabolic reprogramming between naïve and memory B cells: memory B cells carry an elevated heme signature compared to naïve counterparts and retain higher levels of intracellular heme [256]. This maintenance drives increased mitochondrial function, enhancing oxidative phosphorylation and poising memory B cells for plasma cell differentiation [256]. In this way, differences in metabolic programming underlie differences in memory B cell fate in secondary immune responses. In SLE, CD27^+^IgD^+^ memory B cell differentiation into plasmablasts is associated with increased mTORC1 activation and lactate production, indicative of increased glycolysis [257]. Contrastingly, CD27^−^IgD^−^ memory B cells can emerge from CD27^+^IgD^+^ populations with AMP‐activated protein kinase, suppressing mTORC1 and the plasmablast fate [257].

Differences in metabolic programming can divide T‐bet^+^ memory B cells into functionally distinct subpopulations. FcRL5^+^ T‐bet^+^ memory B cells stably upregulate programs related to mitochondrial activity, including oxidative phosphorylation, the electron transport chain and the tricarboxylic acid cycle, in addition to mitochondrial fatty acid β‐oxidation and protein catabolism [175]. This transcriptional program poises this population to rapidly differentiate into antibody‐secreting cells [175]. As such, the proportion of FcRL5^+^ memory B cells in the total memory B pool induced by influenza vaccination correlates with enhanced antibody‐mediated responses post‐boost [175]. Similarly, repeat mRNA vaccination induces two metabolically distinct T‐bet^+^ memory B cell populations: one resembling the FcRL5^+^ population, and the other failing to upregulate the same metabolic pathways [154]. Phenotypically, this second population, expressing the inhibitory receptor CD85j, more closely resembles T‐bet^+^ associated B cells induced by chronic infection than those that contribute to effective vaccine function [22, 147, 148, 149, 154]. In this way, differences in metabolism may help explain the functional heterogeneity of T‐bet^+^ memory B cells.

The success of interferons in establishing antiviral immune responses is, in part, attributed to metabolic rewiring associated with interferon production and receipt. In fibroblasts, macrophages, and dendritic cells, receipt of Type I interferon enhances glucose uptake and enhances glycolysis and glycolysis‐mediated lactate production, while Type II interferon promotes aerobic glycolysis in macrophages [258]. While direct evidence of these metabolic changes has not been observed in B cells, there is evidence that chronic interferon‐α signaling can increase B cell glycosphingolipid expression [259]: a process that appears dependent on glycolytic metabolism and glucose availability [260]. A growing body of evidence supports a role for metabolic programming and the establishment of epigenetic modifications, with several metabolic byproducts directly influencing chromatin accessibility [261]. Logically, it follows that interferon‐driven alterations in metabolism may contribute to differences in memory B cell epigenetic identity in conditions associated with prolonged interferon signaling. While this has not been reported in B cells, in T cells, glycolysis has been shown to drive T‐bet expression [262]: in B cells, a similar mechanism may explain how Type II interferon can promote the expansion of T‐bet^+^ memory B cells.

In altered immune contexts where the memory B cell compartment is remodeled, does reprogramming of B cell immunometabolism drive the emergence of distinct memory B cell populations with different energy requirements? Alternatively, is the emergence of altered memory B cells as an adaptation to sustain the memory B cell pool under microenvironmental conditions that drive metabolic stress? While there is evidence of distinct metabolic states in distinct immune populations, a causative relationship between disease state, immunometabolism, and memory B cells has not been established.

## 6 Concluding Remarks

Memory B cells are an incredibly heterogeneous population, and due to their level of diversity, it is difficult to definitively segregate memory B cells into distinct subsets. Furthermore, memory B cell subsets have been independently identified within different conditions, and published with different nomenclature that makes it difficult to identify commonalities between disease contexts. This notion is further complicated by limited integration of human memory B cell subsets with animal models. A key example of this is the “double negative” memory B cell terminology. While it is a useful classification scheme for human extrafollicular derived memory B cells, it has not been universally adopted. Furthermore, double negative murine B cells in the literature are not equivalent to the human subset they share their name with, in phenotype, fate, or function.

As these subsets continue to be identified, largely through single cell sequencing approaches, it is important to establish universal definitions of different memory B cell subsets. This is critical for the identification of shared populations across multiple disease contexts. By integrating the knowledge obtained from multiple memory B cell datasets, collected from different disease states, common threads can be identified. By exploring these links, further insight can be gained into the drivers of their emergence, thereby unveiling molecular targets that may modulate B cell function. Furthermore, by understanding how cytokines and the disease‐state microenvironment can alter memory B cells, memory B cell remodeling can be considered as a potentially viable therapeutic intervention in dysregulated immune environments.

While memory B cell heterogeneity can complicate the study of memory B cell function, it is also a great immunological strength, with different memory B cell subpopulations and their distributions able to induce varied immune responses across a range of pathologies. Understanding the full extent of this diversity will allow us to fully harness the potential of memory B cells for diagnostic and therapeutic outcomes.

## Funding

This work was supported by the National Health and Medical Research Council, 2033037.

## Conflicts of Interest

The authors declare no conflicts of interest.

## Data Availability

Data sharing not applicable to this article as no datasets were generated or analyzed during the current study.
